# Sarcoma Assessment Measure-Paediatric Version (SAM-Paeds): development of a disease-specific patient reported outcome measure for children with sarcoma

**DOI:** 10.1186/s41687-025-00857-6

**Published:** 2025-03-11

**Authors:** Rachel M. Taylor, Sophie-Anne Purnell, Sian Hocking, Rachael Windsor, Craig Gerrand, Meriel Jenney, Madeleine Adams

**Affiliations:** 1 Centre for Nurse, Midwife and Allied Health Profession led Research (CNMAR), University College London Hospitals NHS Foundation Trust, London, UK; 2 Department of Targeted Intervention, University College London, London, UK; 3 Department of Paediatric Oncology, Noah’s Ark Children’s Hospital for Wales, Cardiff, CF14 4XW UK; 4 London Sarcoma Service, University College London Hospitals NHS Foundation Trust, London, UK; 5 Bone and Soft Tissue Tumour Service, Royal National Orthopaedic Hospital NHS Trust, London, UK

**Keywords:** Sarcoma, Quality of life, Patient-reported outcome, Validation

## Abstract

**Purpose:**

Sarcomas account for approximately 10–15% of all cancer in children aged ≤ 16. Poorer health-related quality of life (HRQoL) is recorded in comparison to other cancers; however, these studies are limited by generic HRQoL measures not being specific to patients with sarcoma. The aim of this study was to develop paediatric version of the Sarcoma Assessment Measure (SAM).

**Methods:**

This mixed methods study comprised three stages: item generation, item reduction and establishing content validity. Children aged 8–16 years and parents of children aged 0–16 years with a diagnosis of sarcoma and within 5 years of completion of treatment were invited to participate.

**Results:**

A total of 29 children and 38 parents from three sites participated in the study. Content analysis of the interview transcripts identified 277 post-diagnosis experience statements of which 128 ‘items’ were included in an Item Reduction Questionnaire, grouped into six domains; physical, disability and inclusion; impact of diagnosis; emotional, impact on family, education. Items with a mean score < 5 and a content validity index of < 0.75 were removed. The final version of SAM-Paeds comprises 33 items (parent version) and 21 items (child version).

**Conclusion:**

This study has developed the first disease-specific HRQoL measure for paediatric sarcoma patients. SAM-Paeds is planned for inclusion within international sarcoma clinical trials and will be validated alongside current generic measures. Developed with the same methodology as the adult SAM questionnaire will facilitate the assessment of QoL longitudinally to assess the long-term impact of the diagnosis and treatment of sarcoma in childhood.

## Abstract (key message)

Sarcoma in childhood causes a wide spectrum of physical and emotional difficulties which are not always captured using the current questionnaires that are used to assess quality of life in childhood cancer patients. In adults, the Sarcoma Assessment Measure (SAM) has been developed specifically for patients with sarcoma. This study explored the experience of children and their families while being diagnosed with and treated for sarcoma and used this information to form a questionnaire (called a patient-reported outcome measure). The Sarcoma Assessment Measure – Paediatric Version (SAM-Paeds), can be used both in the research setting to assess the impact of different treatments on quality of life and in the clinic to highlight patients who need help or interventions to improve their quality of life. The SAM-Paeds questionnaire has two versions, one for children to report themselves and a parent report. SAM-Paeds has more focus on emotional issues such as disability and inclusion as well as long term health effects such as infertility, than the questionnaires that are currently used. Children and families have been involved in all aspects of the study and it is hoped that SAM-Paeds will be a useful tool in both the research and clinical setting.

## Introduction

Childhood cancer has a prevalence of 1:500 children, with sarcomas accounting for approximately 10–15% of cases in children under 16 [1]. Common types include bone sarcomas (Ewing’s and osteosarcoma) and soft tissue sarcomas such as rhabdomyosarcoma. Sarcomas can occur in various body locations, with bone sarcomas often affecting limbs and axial skeleton, and rhabdomyosarcoma found in the head, neck, pelvis, and genitourinary tract. Compared to the excellent survival rates (>80%) for childhood cancers, sarcomas generally have a poorer prognosis, especially in cases with metastatic disease [2].

Treatment for paediatric sarcoma typically involves chemotherapy for systemic disease, along with surgery and/or radiotherapy for local control at the primary site. Due to the varying clinical presentations, disease sites, and treatment modalities, there is a wide range of symptoms and side effects.

The disease itself and the side effects of treatments can lead to significant short and long-term morbidity, extended hospital stays, and adverse effects on the health-related quality of life (HRQoL) of children and their families. HRQoL is measured using patient-reported outcome measures (PROM). In paediatric cancer studies the most commonly used measures are PedsQL 4.0 Generic Core Scales [3], and cancer specific versions [4]. However, there have been limited studies on HRQoL during paediatric sarcoma treatment, with most focusing on small cohorts of patients undergoing specific treatments [5, 6]. In adult studies of HRQoL in cancer the EORTC-QLQ-C30 [7] measure is most commonly used in Europe. The different age-specific PROMS mean that it is not possible to accurately measure changes in HRQoL scores as children transition into adulthood.

There is evidence to suggest that age-associated differences exist in the quality of life of adults living who have been treated for sarcoma with young adults having lower HRQoL scores than older adults, and there is a need to understand the psychosocial impact on the paediatric population [8]. A recent review [9] suggests that generic measures may not capture sarcoma-specific issues, and qualitative research involving young patients is lacking.

The Sarcoma Assessment Measure (SAM) is a PROM that was developed for teenage and adult patients [10, 11]. This study revealed a number of issues not covered by the commonly used generic measures, for example, specific physical issues related to limb sarcomas, disability and rehabilitation as well as emotional concerns such as fear of recurrence. The SAM-Paeds measure has been developed using the same methodology and format as the SAM study, so that the measures can be used to collect longitudinal HRQoL data over time spanning childhood to adulthood.

### Study objectives

1. Explore children and their parent’s sarcoma journey experience from diagnosis to survivorship and use this to form the draft content of the measure (stage 1).
2. Identify the most significant aspects of child/parent experience (stage 2).
3. To draft a test version of SAM-Paeds including the content and response format (stage 2).
4. Confirm content validity of SAM-Paeds (stage 3).
5. Assess the clarity and understanding of the wording of the content in SAM-Paeds (stage 3).

## Methods

### Study design

This was a three-stage, mixed-methods study using semi-structured interviews, focus groups and questionnaires. SAM-Paeds has been developed based on a definition of HRQoL as used in the SAM study: “…subjective, multi-dimensional and dynamic. It is unique to each individual and includes aspects of physical, psychological and social function. It is dependent upon not only the stage of development but also the illness trajectory. This involves the achievement of goals and aspirations and the constraints imposed through ill health and treatment” [10, 12].

### Sample and setting

This study was conducted in three paediatric oncology centres in the United Kingdom (UK). These centres were chosen to ensure a wide demographic was included in the study. The same inclusion criteria were included for all three phases of the study; children aged 8–16 and parents of children aged 0–16 years with a diagnosis of sarcoma, anytime from diagnosis to five years after completion of treatment and ability to communicate verbally or in writing in English either as a native speaker or as a second language. The study was approved by the South-Central Hampshire B Research Ethics Committee (19/SC/0511). An amendment was approved to conduct interviews during phase 1 of the study virtually using video calls, as much of the study was carried out during the COVID-19 pandemic.

In stage 1, the aim was to recruit a purposive sample of 10–20 children and 10–20 parents. Due to the heterogeneity of paediatric sarcoma it was necessary to ensure representation of different ages, disease sites and treatment modalities. Hospital teams identified and obtained written consent from patients and/or parents, which was emailed to the study office. Interviews were not arranged until consent had been received. Participants in stage 1 provided contact details if they wanted to receive information and participate in other stages.

For stage 2, the aim was to recruit 20–40 children and 20–40 parents. Participants were recruited from hospital teams and children who took part in stage 1 were invited by telephone call or email. Children and parents were given information about the study. Both the child and parent versions of the Item Reduction Questionnaire (IRQ) and a freepost return envelope were given to the parents. No identifiable information was included in the IRQ, so parental consent and child assent was implicit through the return of the IRQ. It was advised that children complete questionnaires independently from parents where possible.

In stage 3, children and parents were again identified by hospital teams and patients who provided contact details in stages 1 and 2 were invited to take part in either establishing content validity (10–20 participants) [13] or testing comprehension (10–20 participants) [14]. All materials for establishing content validity were anonymous, so completion was implicit of consent.

### Data collection and analysis

#### Stage 1

Data were collected over a 4-month period, using semi-structured interviews conducted virtually either by telephone or video call. Except for one participant who was interviewed during an inpatient admission for chemotherapy, face-to-face interviews were not possible due to restrictions imposed by the COVID-19 pandemic. Interviews followed a semi-structured schedule developed from the literature [15, 16] and expert opinion (children, parents and healthcare professionals). This was not prescriptive and was purposefully flexible to enable the researcher to explore new and emerging experiences [17]. Interviews were held at a time of the participants’ choice. Where possible children were interviewed independently of their parents, but in some cases a joint interview was conducted at the request of the participants.

All the interviews and focus groups were digitally recorded and transcribed verbatim. Data were analysed using qualitative content analysis to reflect the definition of HRQoL in which the study was based, i.e. “physical, psychological and social function” [14, 18]. Transcripts were reviewed line by line by one researcher (SA–P) and broadly coded into six domains reflecting the impact of diagnosis, physical functioning, emotional, disability and inclusion, effects on family and education. The codes were expanded within each domain to identify its defining characteristics. Quotes and phrases were noted so the items in SAM-Paeds could be based on participants’ actual words. Four other members of the research team independently coded transcripts and discussed discrepancies, relevance and completeness. The research team reviewed the list of generated items and eliminated clearly redundant items through consensus, e.g. items that only occurred in a single interview, did not make clear sense and those which had the same meaning as others. The retained items were formatted for an IRQ to be used in stage 2 of the study.

#### Stage 2

Families received a paper IRQ either given to them during a planned hospital visit or sent in the post to their home address with a freepost return envelope.

The IRQ containing 123 items (child version) and 128 items (parent version) generated from stage 1 of the study were scored according to two factors; “importance” and “worry”. Scores ranged from 1 (least importance/worry) to 5 (most importance/worry) for each item. Data were analysed using SPSS (version 21.0). Mean and median importance and worry scores were calculated and an impact score was calculated as the sum of the importance and worry scales (total score range 2–10). The items rated as most impactful (score ≥ 5) were retained to be reviewed for relevance by children/parents and healthcare professionals. These items were grouped within overarching categories (themes) by the research team.

#### Stage 3

##### Establishing content validity

The Content Validity Questionnaire (CVQ) included all the items for each domain of the questionnaire retained after stage 2 and respondents were instructed to review all the items and rate their relevance to the measure as a whole on a scale of 1 (not relevant) to 4 (highly relevant) [18, 19]. Participants were sent a copy of the Content Validity Questionnaire (CVQ) either as a paper questionnaire or as an online version according to preference. Paper copies of the CVQ were handed to research staff or returned in a stamped addressed envelope.

Data were entered into SPSS (version 21.0); data from healthcare professionals, children and parents were analysed separately. The content validity ratio (CVR) is an item-level statistic to identify items to retain or reject. The CVR was calculated for each item using the formula: *ne* N2/N2, where *ne* was the number of respondents who rated the item as relevant/highly relevant and N was the total number of respondents. Based on a sample size of 10 all items with a score lower than 0.75 were discarded; the bigger the sample, the lower the CVR that could be accepted [20]. The content validity index (CVI) was calculated through computing the mean CVR of all the retained items. Content validity was confirmed with a CVI > 0.75 for the parent version and 0.78 for the child version (variation reflecting the greater number of parent participants than children) [20, 21].

##### Testing comprehension

Comprehension of the questionnaire was confirmed through cognitive interviews conducted by one researcher (MA). Participants were shown a copy of SAM which included the final 33 items (parent version) or 21 items (child version). Questions were asked to test various aspects of comprehension (interpretation, need for clarification, misunderstanding, memory/recall) and response. Notes were made and discussed with the research team and modifications to wording of questions were made.

## Results

Patient characteristics for all stages of the study are included in Table 1. There were a higher proportion of younger patients aged less than 8 years with rhabdomyosarcoma and pelvic and head and neck sites.

**Table 1:** Participant characteristics of all stages

Demographic		Child*	**Parent only	Total number n = 29	Percentage
Gender	Male	9	5	14	48
	Female	12	3	15	52
Age (years)	0–8	0	8	8	28
	8–16	21	0	21	72
Diagnosis	Osteosarcoma	14	0	14	48
	Ewing’s sarcoma	4	1	5	17
	Rhabdomyosarcoma	2	5	7	24
	Other	1	2	3	10
Disease site	Head and neck	2	2	4	14
	Extremity/limbs	15	1	16	66
	Pelvis	3	3	6	21
	Other	1	2	3	10
Treatment	Surgery alone	2	1	3	10
	Chemotherapy and surgery	15	2	17	59
	Chemotherapy, surgery and radiotherapy	4	5	9	31
Parent participants (n = 38)	Mother			28	74
	Father			10	26

*Number of patients where child could participate themselves, **number of patients where parents responded on behalf of child (all were aged less than 8 years old)

### Stage 1

A total of 20 participants (12 parents and 9 children) consented to participate in stage 1 of the study. One parent and one child were interviewed face-to-face while the child was an inpatient for chemotherapy and the other participants were interviewed virtually via video call. Content analysis of the parent alone or parent and child combined interviews revealed 237 items. An additional 40 items were identified from the interviews with children alone. These items were grouped into six domains to reflect families sarcoma experience; physical (n = 78), impact of diagnosis (n = 38), emotional (n = 59), disability and inclusion (n = 33), social/impact on family (n = 45) and education (n = 27). The experiences described, reflected specific issues of children with sarcoma and their parents including surgical scars (“my scar reminds me of my time in hospital”), amputation (“I am very sporty so we decided amputation was best”), fear of recurrence (“I do worry when I have scans, that the cancer could come back”) and risk of infertility (“because it was a lot in his pelvis treatment, we are still waiting to find out about fertility…. to see if there has been any lasting damage”).

The items were taken from each individual interview to keep them close to the patient’s words, so initially there were multiple items about the same issue, e.g. fear of recurrence; patients described it using slightly different expressions so they were grouped together and through research team discussion, the item that best represented the topic was selected. Some items that were deemed to be merely descriptive were removed. All the relevant topics were kept for the next stage. This was done through extensive and iterative review of the items, involving all members of the research team until 128 items (parent version) and 123 items (child version) were retained to be included in the IRQ used in stage 2.

### Stage 2

A total of 16 parents and 13 children completed the IRQ; 46/123 (37%) and 59/128 (48%) items had a mean impact score of ≥ 5 in the child and parent questionnaire respectively. Table 2 summarises the scores per domain.

**Table 2:** Number of items with a mean impact score ≥ 5

Domain	Parent IRQ n = 128				Child IRQ n = 123			
	N	Mean impact score	n ≥ 5	% ≥ 5	N	Mean impact score	n ≥ 5	% ≥ 5
Physical	6	6.0	5	83	9	5.4	7	78
Impact of diagnosis/treatment	26	5.4	16	61	18	4.7	8	44
Emotional	26	5.7	22	84	30	5.3	22	73
Disability and inclusion	35	4.5	10	29	32	4.0	3	9
Social/family impact	17	4.7	5	29	13	4.3	2	15
Education	18	4.0	1	6	21	4.5	4	19

Following analysis of the IRQ, 13 and 22 items that were duplicate or similarly worded were removed, leaving 33 questions in the child version and 37 in the parent version of the SAM-Paeds for use in stage 3. Within the child version five questions that were felt to be important to assess the impact of the service were retained. Due to small numbers of questions within the disability, social and education domains, these were grouped together leaving three domains (physical, emotional, and social functioning). The items in the domains in SAM-Paeds therefore included six physical, 16 emotional, 11 social and five service-related items for the child version, and seven physical, 22 emotional and eight social items for the parent version, which were included in the CVQ in stage 3.

### Stage 3

Eight parents and nine children completed the CVQ; the CVR (based on sample size), required to retain an item was therefore 0.75 and 0.78 respectively. Analysis of the results demonstrated that all items within the parent questionnaire achieved a cut off score of ≥ 0.75. Ten items were removed from the child version as they failed to reach the cut off score of ≥ 0.78. Subsequent review identified two items from the child version and four from the parent version that were deemed generic and were covered within other widely used HRQoL measures (PedsQL™ [3] and EORTC-QLQ-C30 [7]) and were therefore removed.

**Fig. 1:**
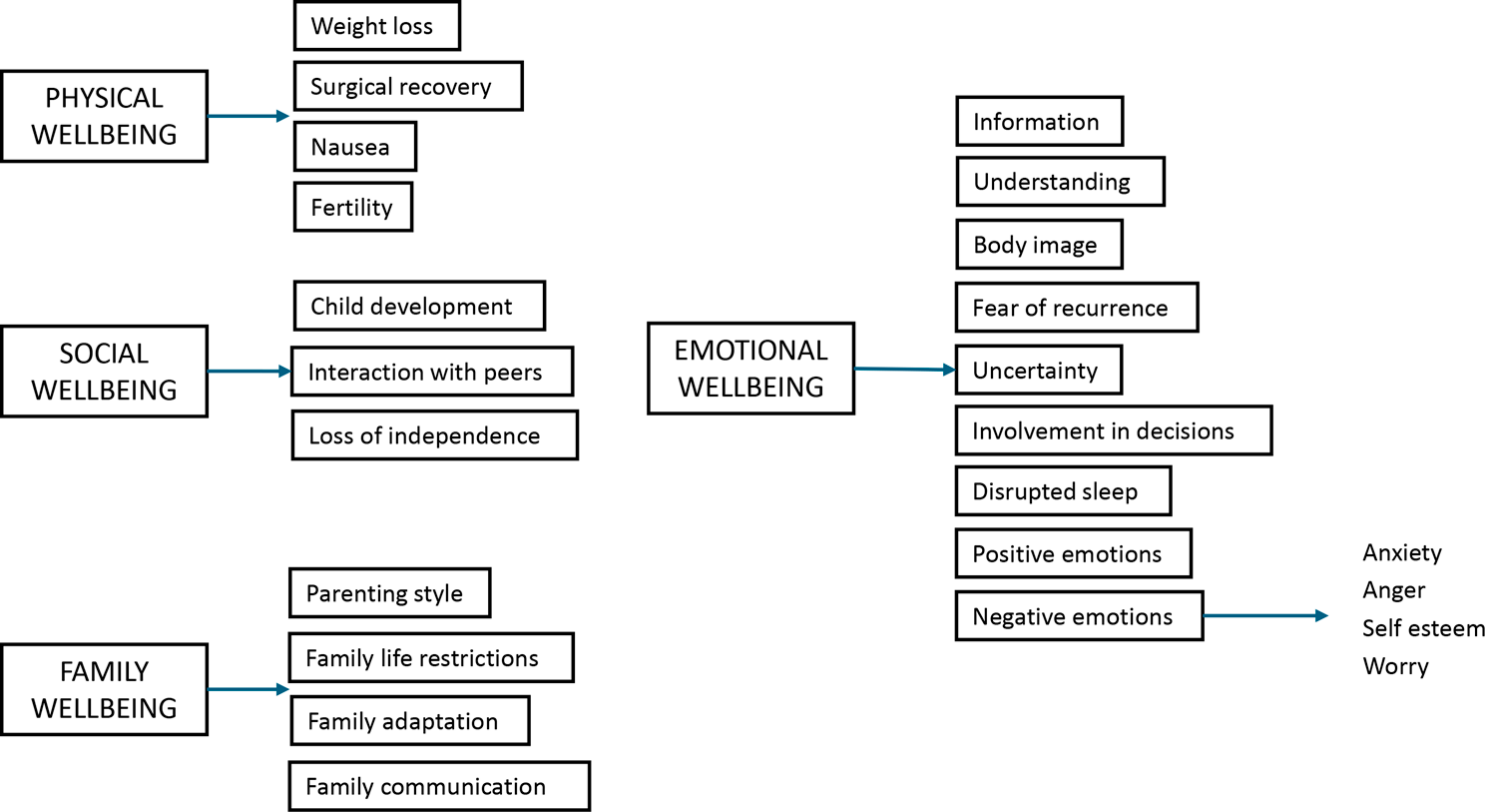
Summary of the structure and content of the parent proxy report

**Fig. 2:**
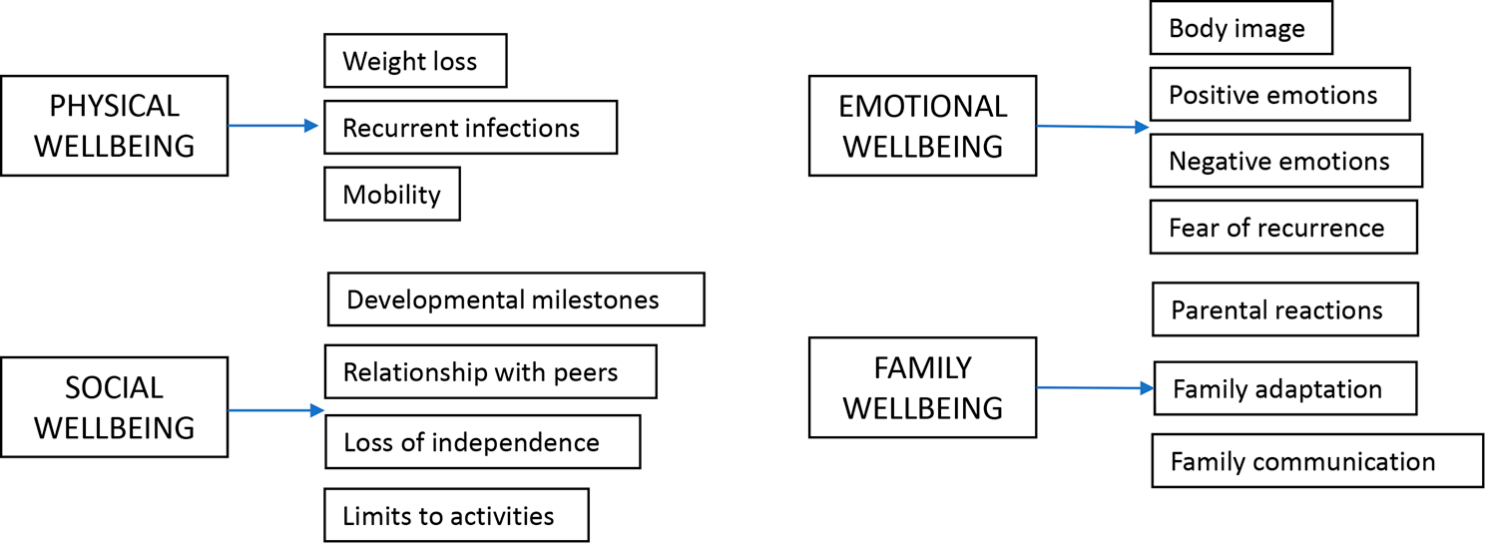
Summary of the structure and content of the child self-report

**Fig. 3:**
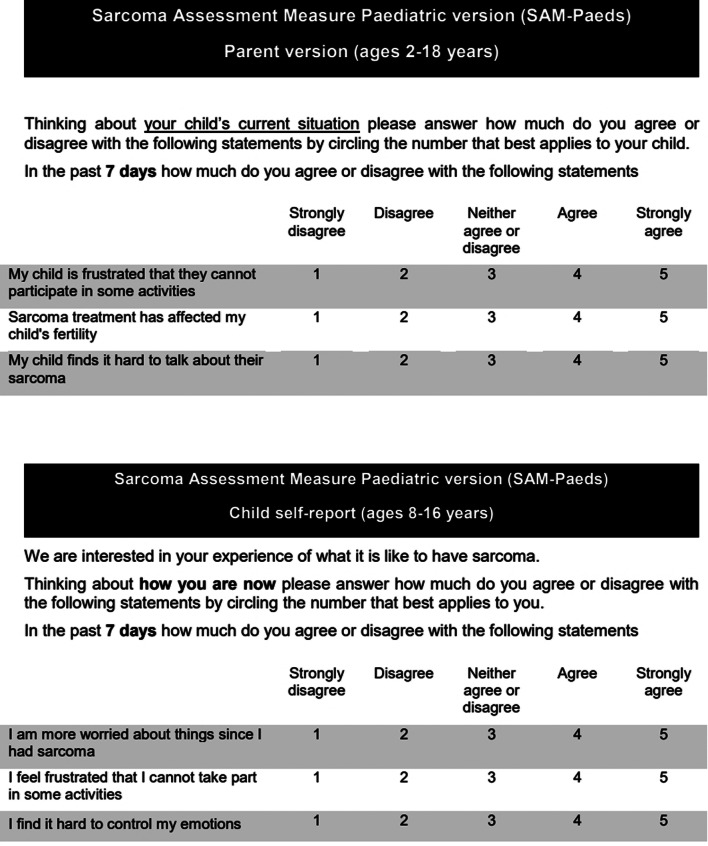
Layout and example questions of SAM-Paeds parent and child version

Six children and eight parents completed the questionnaire alongside the researcher (MA) to test comprehension. Following this, minor changes to the wording were made. The prototypes of the SAM-Paeds questionnaire therefore included 33 items across four domains for the parent proxy version (Fig. 1) and 21 in the child self-report (Fig. 2). Layout and example questions are shown in Fig. 3.

## Discussion

The aim of SAM-Paeds was to create a Patient reported outcome measure (PROM) that can be used for children with sarcoma irrespective of sarcoma sub-type, disease site and treatments received. The formatting and scoring system was designed to complement the adult SAM questionnaire, to allow longitudinal measurement of the impact of living with sarcoma as patients transition from being children, teenagers and into adulthood. The measure is designed to be used both in clinical practice as well as in the clinical trial settings as part of PROM data collection. The final version of SAM-Paeds has 21 questions in the child self-report (8–16 years) and 33 questions in the parent-proxy version.

This paper describes the development of SAM-Paeds, a disease-specific, PROM reflecting the experience of children affected by sarcoma and their families. PROMs are recognised as the gold standard for measuring HRQoL of children and families in paediatric oncology [22]. The importance of developing measures for specific subtypes of childhood cancer is increasingly recognised. Large cohort studies on long-term survivors of childhood cancer have found survivors of soft tissue sarcoma had lower overall physical HRQoL compared to other survivors of childhood cancer [23]. Similar results are also seen in survivors of bone sarcomas [24]. Currently, PROM use in paediatric sarcoma clinical care and research is relatively scarce and symptoms are often graded by healthcare professionals using tools such as the common toxicity criteria for adverse events (CTCAE) [25] who base their assessment mostly on observation [26]. This is problematic, especially for less visible symptoms, because it has been demonstrated that agreement between children, parent and clinician’s grading is low, and clinicians consistently underreport children’s symptoms [27, 28]. While there is a patient reported version of the CTCAE (PRO-CTCAE), this has not been specifically validated for children with sarcoma [29].

Sarcoma patients are different to those with other cancer types for several reasons; firstly, it can occur at any age from early childhood, adolescence and into older adulthood and secondly due to its relative rarity, healthcare knowledge on sarcoma is limited and so accessing information and support can be difficult. In addition, the disease and treatment can result in physical and disabling consequences that occur and change long after the initial treatment (particularly for the very young child), meaning that the impact of the late effects of treatment for patients changes over time and patients will require long term access to healthcare (including transition from child to adult services). By involving patients reflecting the range of sarcoma subtypes, site, ages and treatment trajectory and geographical locations in the UK, we have been able to identify key issues for children and their families with sarcoma.

As with the development of any PROM, there was the concern that SAM-Paeds would be too similar to other already validated generic HRQoL measures. However, most of these measures are heavily weighted towards physical aspects of HRQoL whereas in SAM-Paeds, the majority of items reflect emotional (60% in parent version, 38% in child version) and social issues (24% in parent version and 42% in child version). Only 15% of items in the parent version and 19% of items in the child version relate to physical issues and those are related to issues such as fertility and weight loss, rather than fatigue or pain as seen more commonly in generic measures. This is similar to the SAM study where 68% of the items were within the emotional domain [10]. The difference between the parent and child version of SAM-Paeds are mostly reflective of a parent’s anxiety around the wellbeing of their child and the child’s focus on social inclusion as being important to them.

Similar themes were identified in the adult SAM measure although there was more focus on physical symptoms for patients with limb sarcoma. The differences seen between the SAM and SAM-Paeds studies, could be accounted for by the relatively lower frequency of extremity sarcomas in children or it may be that children focus less on physical restrictions and more on social interactions and peer relationships.

Our study was limited as we used a convenience sample of children and parents presenting in three specialist sarcoma centres. While these reflected the types of sarcomas, presenting in childhood, it does not reflect the full range of subtypes and therefore the experiences of children with rarer types of sarcomas might not be represented. Despite this limitation, these subtypes are rare and the participants in our study reflect the majority of the paediatric sarcoma population in the UK. As this is the first study to develop a sarcoma-specific PROM for children, this should be the prototype, in which future validation studies are based.

## Conclusion

This study has developed the first disease-specific HRQoL measure for paediatric sarcoma patients. Developed with the same methodology as the adult SAM, SAM-Paeds has been developed for use in clinical practice to highlight patients who would benefit from interventions such as psychosocial support. The prototype of SAM-Paeds requires more extensive validation. Sarcoma is a rare cancer type and therefore recruiting a sufficiently large sample of children and parents to be able to undertake this validation is a challenge. As a first step, SAM-Paeds is planned for inclusion, alongside generic PROMs within a number of international paediatric sarcoma clinical trials. Further studies are also planned to evaluate longitudinal data collection using the SAM-Paeds and TYA/adult SAM measures.

## Data Availability

The datasets used and/or analysed during the current study are available from the corresponding author on reasonable request.

## Abbreviations

CTCAE: Common toxicity criteria for adverse events
CVI: Content validity index
CVQ: Content validity questionnaire
CVR: Content validity ratio
HRQoL: Health-related quality of life
IRQ: Item Reduction Questionnaire
PRO-CTCAE: Patient-reported out of common toxicity criteria for adverse events
PROM: Patient-reported outcome measure
SAM: Sarcoma Assessment Measure
SAM-Paed: Sarcoma Assessment Measure Paediatric Version
